# Long-term outcomes of augmented unilateral recess-resect procedure in children with intermittent exotropia

**DOI:** 10.1371/journal.pone.0184863

**Published:** 2017-10-06

**Authors:** Jin-Soo Kim, Hee Kyung Yang, Jeong-Min Hwang

**Affiliations:** Department of Ophthalmology, Seoul National University College of Medicine, Seoul National University Bundang Hospital, Seongnam, South Korea; Cairo University, EGYPT

## Abstract

**Background:**

Initial overcorrection after exotropia surgery has been considered as a desirable result. Recently, there had been several studies that reported better surgical results of augmented bilateral lateral rectus muscle recession procedure over the conventional procedure.

**Objectives:**

To compare the long-term results of augmented unilateral lateral rectus recession-medial rectus resection procedure (RR) with the original surgery in exotropic children.

**Data extraction:**

A retrospective cohort study was performed on a total of 121 children with exotropia who underwent RR from February 2005 to December 2012 and were followed-up for at least 24 months. In 64 patients, RR was performed based on the original surgical table (original RR group). In 57 patients, the amount of medial rectus muscle resection was increased by 1 mm (augmented RR group).

**Results:**

In the original RR group, 47 of 64 patients (73.4%) had a successful outcome, 13 patients (20.3%) had recurrence, and 4 patients (6.3%) had overcorrection at 2 years after surgery. In the augmented RR group, 45 of 57 patients (79.0%) were successful, 4 patients (7.0%) had recurrence and 8 patients (14.0%) had overcorrection at 2 years after surgery. The recurrence rate was significantly lower in the augmented RR group than the original RR group, whereas the overcorrection rate was not significantly different between two groups at 2 years after surgery (*P* = 0.036 and *P* = 0.153, respectively). The cumulative probability of recurrence was lower in the augmented group at 36 months after surgery (*P* = 0.046, log rank test).

**Conclusions:**

The long-term success rate of augmented RR in exotropic children was 79.0% and the recurrence rate was significantly lower than original RR with comparable overcorrection rates. Augmented RR can be considered as an alternative procedure in children with basic and convergence insufficiency type exotropia.

## Introduction

Bilateral lateral rectus muscle recession (BLR) and unilateral lateral rectus recession-medial rectus resection (RR) have been the two most popular surgical techniques for exotropia.[1–8] There had been many studies comparing BLR and RR in intermittent exotropia showing various results.[1–8] RR tended to have better success rates compared to BLR until 1.3 years and lower success rates after 2 years, but overall surgical outcomes were comparable.[1, 2, 5] Choi et al [5] showed better final outcomes of BLR over RR, since RR showed continuous recurrence of exotropia after postoperative 6 months. They suggested that a gradual loss of fusion by incomitance in horizontal gaze and decrease of the tethering effect of the medial rectus muscle by muscle stretching was the reason for continuous recurrence of exotropia after RR. Augmentation of the amount of medial rectus resection might overcome such decrease of the tethering effect. However, to the best of our knowledge, no report has been published comparing surgical outcomes of augmented RR procedure in exotropia patients.

Initial overcorrection after exotropia surgery has been considered as a desirable result since postoperative exodrift was common in many observational studies.[1, 5, 8, 9] Although some studies reported that initial overcorrection cannot predict long-term motor outcomes,[10, 11] other studies reported that initial overcorrection was a predictor of a successful long-term outcome after surgery.[9] Recently, there had been several studies that reported better surgical results of augmented BLR procedure over the conventional BLR procedure.[12, 13] These studies reported reduced recurrence rates by increasing the amount of lateral rectus muscle recession. Studies comparing surgical results in convergence insufficiency-type exotropia showed that the RR procedure was significantly more successful than the BLR procedure.[14, 15] In these studies, the amount of medial rectus resection was based on the near deviation and amount of lateral rectus recession was based on the distant deviation in the RR group.[14, 15] In our study, we evaluated the long-term surgical outcomes of augmented RR by increasing the amount of medial rectus muscle resection, and compared the results with the original procedure.

## Methods

A retrospective review of medical records was performed on randomly selected 288 children between 3 and 10 years-old who underwent RR procedure for intermittent exotropia by 1 surgeon (J-M.H) between February 2005 and December 2012. The minimum required follow-up period after surgery was 24 months, except for patients who required reoperation within 24 months after the primary surgery. Surgical outcomes at 3 years after surgery were also assessed in those who had available data. Patients with paralytic or restrictive strabismus, true divergence excess type intermittent exotropia, preoperative deviation > 50 prism diopters (PD), anisometropia, developmental anomalies, neurologic disorders, history of previous strabismus surgery, moderate to severe amblyopia, coexisting ocular diseases other than strabismus, and infantile exotropia were excluded. Patients with dissociated vertical deviation, A or V patterns, simulated divergence excess type intermittent exotropia or oblique muscle overactions not requiring surgery were included. This study adhered to the Declaration of Helsinki and the protocol was approved by the institutional review board of Seoul National University Bundang Hospital. All patient records were de-identified and analyzed anonymously.

### Preoperative ophthalmologic examination

All patients underwent a complete preoperative ophthalmologic examination including prism and alternate cover testing with accommodative targets for fixation at 1/3 and 6 m. In case of distant deviation exceeding near deviation by 10 PD or more, an additional near measurement was obtained after 1 hour of monocular occlusion of the habitually deviating eye. True divergence excess-type exotropia was defined when this difference kept exceeding 10 PD even after occlusion. Convergence insufficiency-type exotropia was defined when near deviation exceeded distance deviation by 10 PD or more.

Refractive errors were measured by cycloplegic refraction and analyzed as spherical equivalent values. Anisometropia was defined as a spherical equivalent difference > 1.50 diopters (D) between both eyes. Amblyopia was defined as a difference of 2 lines or more between monocular visual acuities and only mild amblyopia with a difference of 2 lines were included. Stereopsis was evaluated with the Randot stereoacuity test at distance and near. Stereopsis of ≤ 100 seconds of arc was defined as good.

### Intraoperative procedures

All surgeries were performed under general anesthesia by one surgeon (J-MH). All patients underwent RR on the nondominant eye, if present, based on the largest angle of preoperative deviation measured at distance or near.[16] In the original RR group, those who underwent surgery between February 2005 and August 2008, the surgical dose was determined according to the Wright formula.[17] In the augmented RR group, those who underwent surgery between May 2012 and December 2012, the amount of medial rectus muscle resection was increased by 1 mm regardless of the preoperative angle of deviation (Table 1).

**Table 1:** Surgical table.

Group	Deviation at distance, PD	LR recession, mm	Deviation at near, PD	MR resection, mm
Original RR	15	4	15	3
	20	5	20	4
	25	6	25	5
	30	7	30	5.5
	35	7.5	35	6
	40	8	40	6.5
	50	9	50	7
Augmented RR	15	4	15	4
	20	5	20	5
	25	6	25	6
	30	7	30	6.5
	35	7.5	35	7
	40	8	40	7.5
	50	9	50	8

PD = prism diopters, LR = lateral rectus muscle, MR = medial rectus muscle, RR = lateral rectus muscle recession and medial rectus muscle resection

### Postoperative measurements

Postoperative assessments were made at 1, 6 months, and 2 years after the operation. Assessment at 3 years after surgery was also made for those who were available.

Postoperative examinations were taken in the same manner as the preoperative examinations. The eye that did not undergo surgery underwent full-time occlusion until 1 month after surgery. If consecutive esotropia persistently existed at 1 month after the operation, hyperopia> +1.00 D was corrected and base-out Fresnel press-on prisms (3M Health Care, St Paul, Minnesota, USA) were prescribed to facilitate constant fusion. Prism-incorporated regular spectacles were prescribed in case it was evident that prisms would have to be worn for several months.

Surgical success was defined as a postsurgical alignment between 10 PD of esodeviation and 10 PD of exodeviation at distance and near in the primary position. Recurrence was defined as a postsurgical alignment of > 10 PD of exodeviation, and overcorrection defined as > 10 PD of esodeviation at distance and/or near. Reoperation for overcorrected patients was performed if esotropia ≥ 20 PD persisted or increased over 6 months after surgery. Reoperation for recurrent or residual exotropia was performed in constant exotropia ≥ 14 PD at distance, despite any nonsurgical treatments, such as part-time occlusion or minus-lens therapy. Improved stereopsis was defined as a decrease of 2 octaves or more and decreased stereopsis was defined as an increase of more than 2 octaves or more.[18]

### Main outcome measures

Primary outcome measures were surgical success rates at 2 years after surgery and improvement in stereopsis. Secondary outcome measures were risk factors for recurrence and overcorrection. Age, sex, angle of deviation, amount of surgery and type of exotropia were evaluated for possible risk factors for overcorrection and recurrence of exotropia.

### Statistical analysis

The student’s *t*-test, chi-square test, Fisher’s exact test and Mann-Whitney test were used to compare the patient characteristics and the surgical outcomes. Kaplan-Meier survival analysis and the log rank test were used to compare the long-term cumulative probability of recurrence between both groups. P value of < 0.05 was considered as statistically significant. All statistical analyses were performed with the SPSS software for Windows (version 21.0; SPSS Inc., Chicago, Illinois, USA).

## Results

### Preoperative patient characteristics

Among the 192 children between 3 and 10 years-old who underwent RR surgery between 2005 and 2012 and were followed-up for more than 24 months, 71 patients who did not meet the eligible inclusion criteria were excluded. A total of 64 patients in the original RR group and 57 patients in the augmented RR group were finally included in analyses. The preoperative patient characteristics were not significantly different between the two groups except for the amount of medial rectus muscle resection (Table 2). The maximum preoperative angle of deviation at distance was 27.4 ± 8.3 PD in the original RR group and 27.6 ± 6.8 PD in the augmented RR group (*P* = 0.905). Thirty-seven patients in the original RR group (68.5%) and 38 patients in the augmented RR group (71.7%) had good stereopsis, which was not significantly different between both groups (*P* = 0.548). Eight patients in the original RR group (12.5%) and 10 patients in the augmented RR group (17.5%) had mild amblyopia, which was also not significantly different between both groups (*P* = 0.436).

**Table 2:** Demographics.

	Original RR	Augmented RR	P—value
No. of patients	64	57	
Mean age at surgery (years)	6.0 ± 2.3 (3–10)	5.9 ± 1.9 (3–10)	0.822^c^
Sex (Male/Female)	31 / 33	31 / 26	0.513^a^
Total follow-up (months)	32.8 ± 10.9 (6–43)	32.9 ± 8.3 (7–43)	0.958^c^
Classification of exotropia			0.868^b^
Basic	59 (83.08%)	53 (84.48%)	
Convergence insufficiency	5 (7.70%)	4 (6.90%)	
Preoperative deviation (PD)			
At distance	27.4 ± 8.3 (14–50)	27.6 ± 6.8 (14–40)	0.905^c^
>30 PD at distance	13 (20.31%)	15 (26.32%)	0.434^a^
At near	28.0 ± 8.4 (14–40)	28.6 ± 7.8 (14–40)	0.668^c^
>30 PD at near	16 (25.00%)	20 (32.09%)	0.226^a^
Amount of surgery (mm)			
LR recession	6.6 ± 1.0 (4–8)	6.4 ± 1.1 (4–8)	0.262^c^
MR resection	5.5 ± 0.7 (4–7)	6.2 ± 1.0 (4–7.5)	<0.001^c^
Mild amblyopia	8 (12.5%)	10 (17.5%)	0.436^a^
Good stereopsis	37/54 (68.5%)	38/53 (71.7%)	0.548^a^

^a^Chi-square test,

^b^Fisher's exact test,

^c^Independent t-test,

RR = lateral rectus recession and medial rectus resection, PD = prism diopters, LR = lateral rectus muscle, MR = medial rectus muscle

### Surgical outcome

**Fig 1:**
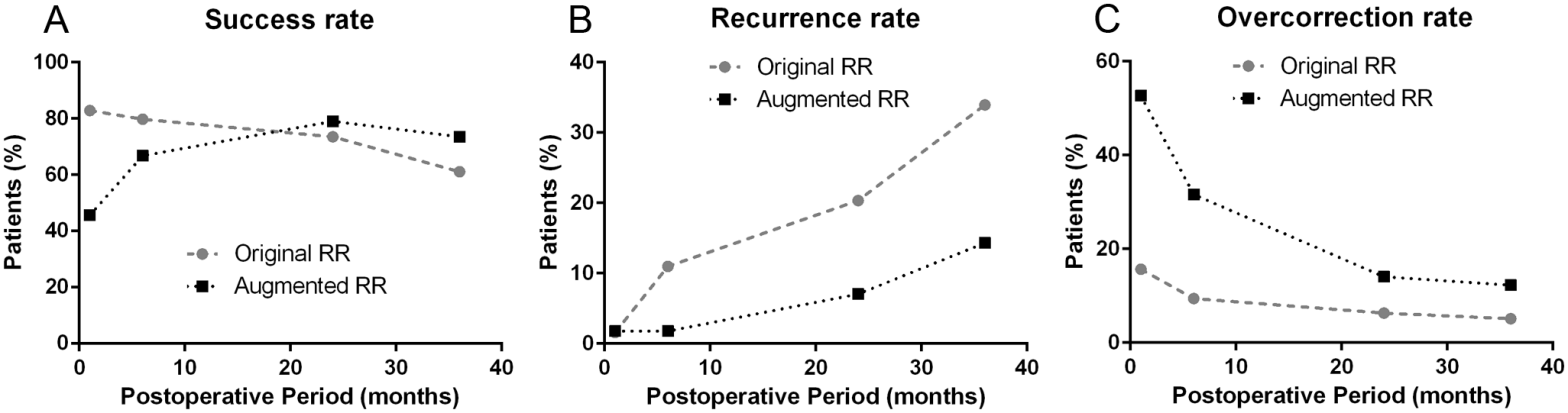
Surgical outcomes. Percentage of patients resulting in success (**A**), recurrence (**B**) and overcorrection (**C**) at 1 month, 6 months, 2 years and 3 years after the original unilateral lateral rectus recession-medial rectus resection (RR) and augmented RR.

In the original RR group, 47 of 64 patients (73.4%) were successful, 13 patients (20.3%) had recurrence, and 4 patients (6.3%) had overcorrection at the 2-year follow-up examination. In the augmented RR group, 45 of 57 (79.0%) patients were successful, 4 patients (7.0%) had recurrence, and 8 patients (14.0%) were overcorrected (S1 Table, Fig 1). Recurrence rate was significantly higher in the original RR group at 2 years after surgery (*P* = 0.036) and this continued up to 3 years after surgery (*P* = 0.019). Overcorrection rate was significantly higher in the augmented RR group compared to the original RR group up to 6 months, but the gap continued to decrease and finally, the overcorrection rate was not significantly higher at 2 years (*P* = 0.153) and 3 years after surgery (*P* = 0.180).

The mean time to recurrence after operation was 26.5 ± 7.2 months in the original RR group (n = 20) and 23.6 ± 13.3 months in the augmented RR group (n = 7), which was comparable between two groups (*P* = 0.337, Mann-Whitney test). Twenty-two patients required reoperation for recurrence, 15 patients (23.4%) in the original RR group and 7 patients (12.3%) in the augmented RR group, but there was no significant difference in the percentage of reoperation between both groups (*P* = 0.104, Fisher’s exact test).

Two patients (3.1%) in the original RR group and no patient (0%) in the augmented RR group required reoperation for overcorrection. In those two patients, the time to reoperation for consecutive esotropia after the initial surgery was 6 and 7 months, respectively.

**Fig 2:**
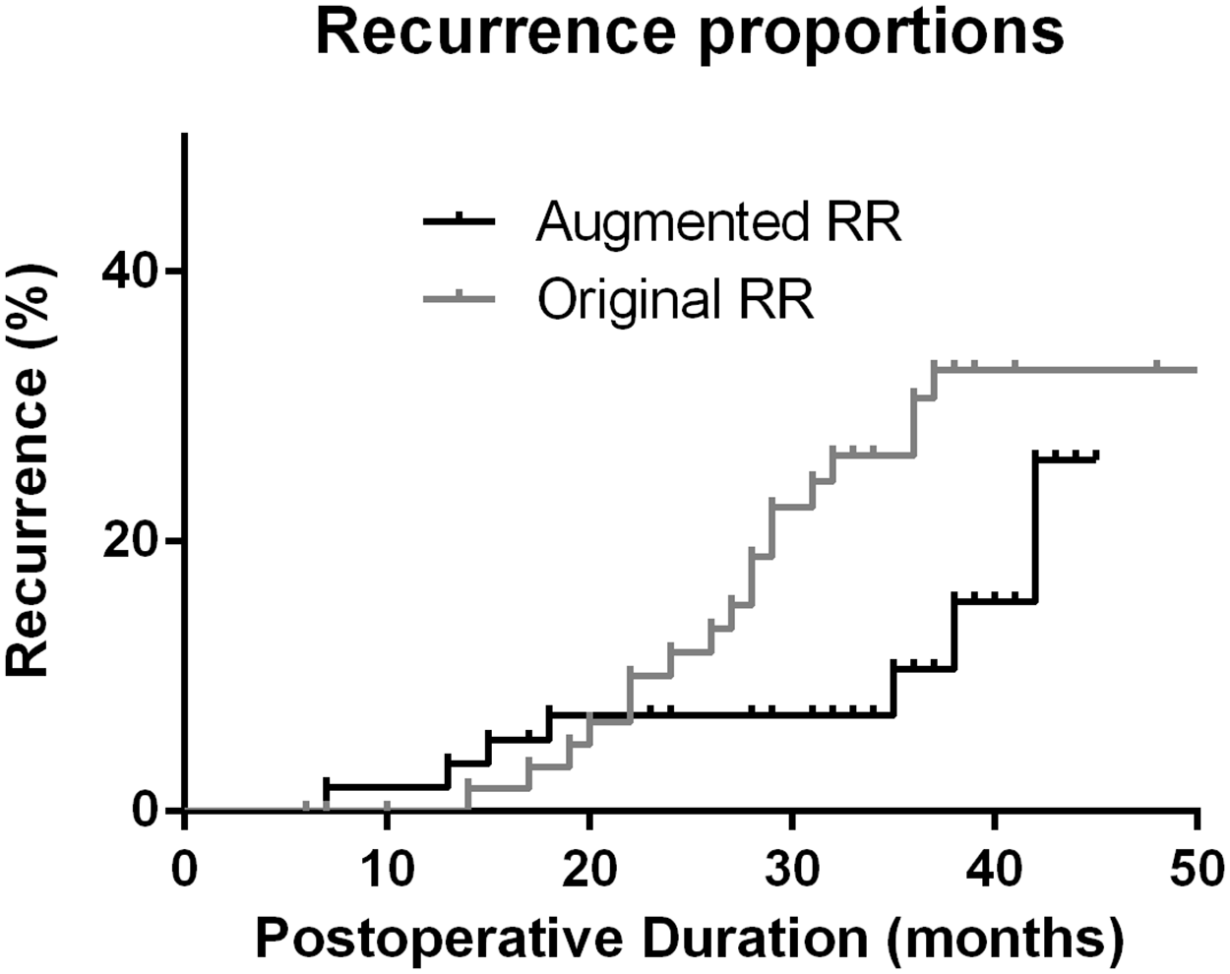
Survival curves. Kaplan-Meier survival curves after the original unilateral lateral rectus recession-medial rectus resection (RR) and the augmented RR. The cumulative probability of recurrence was lower in the augmented RR group at 36 months after surgery (*P* = 0.046, log rank test).

By Kaplan-Meier survival analysis, the cumulative probability of recurrence was lower in the augmented group (24.6%) compared to the original RR group (42.2%) at 36 months after surgery (*P* = 0.046, log rank test, Fig 2). The rates of recurrence per person-year were 11.5% in the original RR group and 4.6% in the augmented RR group.

### Binocular function (stereopsis)

Fifty-four patients in the original RR group and 57 patients in the augmented RR group were capable to test stereoacuity at 2 years after surgery. Good stereoacuity was present in 70.4% (38/54) in the original RR group and 73.7% (42/57) in the augmented RR group (*P* = 1.000, chi-square test) at the 2-year follow-up examination. Improvement in stereopsis was found in 34.0% (18/53) in the original RR group and 37.7% (20/53) in the augmented RR group, whereas decreased stereopsis was found in 11.3% (6/53) in the original RR group and 5.7% (3/53) in the augmented RR group. The sensory outcome was not significantly different between both groups (*P* = 0.647, Fisher’s exact test).

### Risk factors for recurrence or overcorrection

Recurrence at 1 month after surgery was the only significant risk factor for recurrence at 2 years after surgery by univariate and multivariate analysis of variance. Overcorrection at 1 month after surgery was the only significant risk factor for overcorrection at 2 years after surgery by univariate and multivariate analysis of variance.

### Postoperative exodrift

The exodrift rate between postoperative 1 month and 2 years was 2.8 ± 3.4 PD/year at distance in the original RR group and 3.0 ± 4.0 PD/year at distance in the augmented RR group. There was no significant difference between two groups (*P* = 0.716). The exodrift rate between postoperative 1 month to 6 months and 6 months to 2 years also showed no significant difference between both groups at distance and near (S2 Table). The exodrift rate was significantly slower after 6 months in both groups at distance and near.

### Postoperative complication

There were no patients with noticeable abduction or adduction deficits. No patients complained of definite asymmetric lateral incomitance in both group. Additionally, there were no other postoperative complications.

## Discussion

In this study, we performed an augmented RR procedure which increased the amount of medial rectus muscle resection by 1 mm in intermittent exotropia children, and compared the surgical outcomes with the original RR procedure. First, the augmented RR showed more successful long-term results than the original RR at 36 months after surgery. Second, despite the concerns of overcorrection, the overcorrection rate in the augmented RR was comparable to that of the original RR.

Although consecutive esotropia generally recovers over time, persistent overcorrection may be associated with the risk of amblyopia and loss of stereopsis in young children.[19] In our study, however, sensory outcomes were not different between both groups despite the higher rate of overcorrection rate in the augmented RR group during the early postoperative period. A previous study by our group [20] suggested that prismatic correction can lead to good motor outcomes while maintaining favorable sensory status in most patients with consecutive esotropia. We changed our surgical table since 2012 in order to improve surgical outcomes while preserving binocular sensory fusion.

Previous studies showed that success rates of RR procedure for intermittent exotropia were 33% to 83%.[1–3, 21, 22] Although these studies had variable definition of success and follow-up periods, most of the surgical failure was due to recurrence of exotropia. In the augmented RR group in our study, high overcorrection rates resulted in lower success rates at postoperative 1 month. However, overcorrection rates decreased over time and the overall success rates were not significantly different in the two groups from 6 months to 3 years after surgery. Cumulative probability of recurrence was lower in the augmented RR group at 36 months after surgery. Recurrence rates were also significantly lower in the augmented RR group at postoperative 2 and 3 years. The recurrence rate of 7.0% at postoperative 2 years is remarkably low compared to previous studies, which ranged between 37.7% and 60.7%.[3, 5–7, 23]

**Fig 3:**
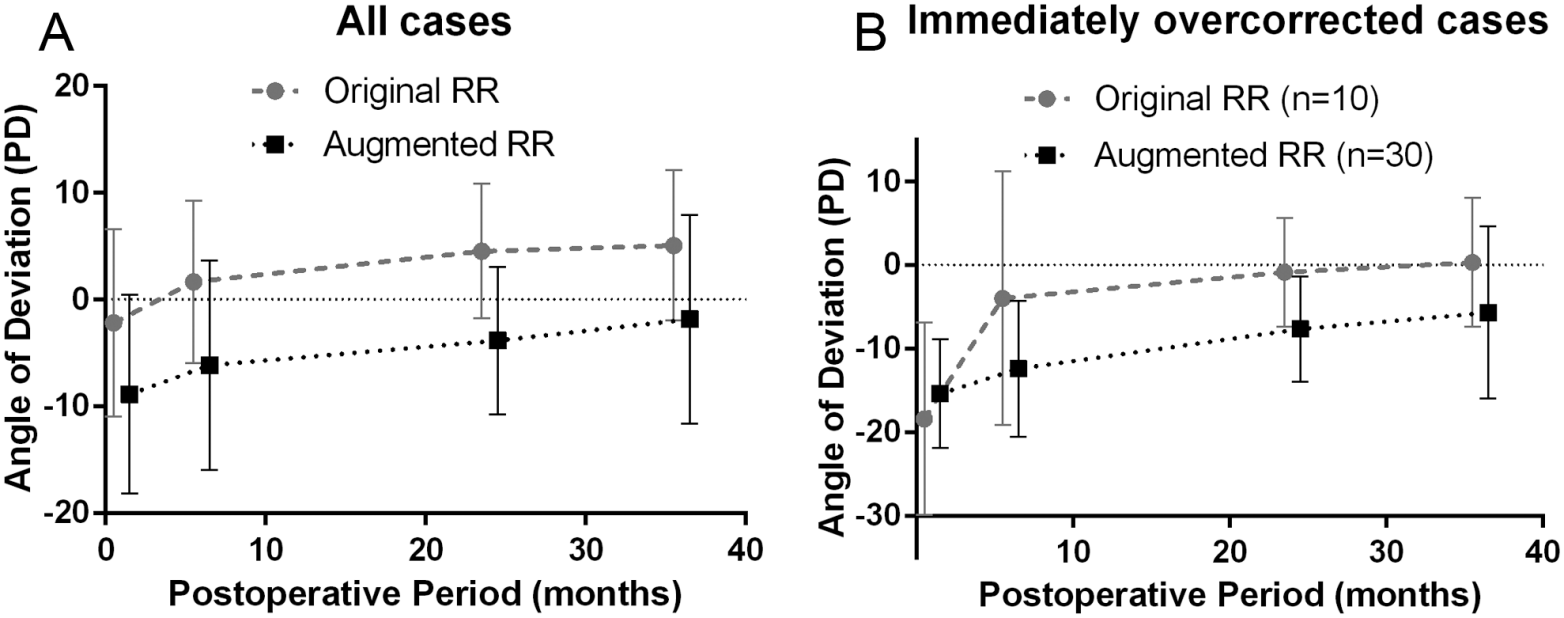
Exodrifts. The mean angle of deviations at each postoperative period after the original unilateral lateral rectus recession-medial rectus resection (RR) and the augmented RR. In all enrolled cases (**A**), the exodrift rates of the two groups were almost parallel throughout the observation period and showed a continuous decrease after surgery. In overcorrected cases at 1 month after surgery (**B**), exodrift rates also showed a continuous decrease after surgery. PD = prism diopters.

Exodrift rates were not significantly different between the two groups throughout the postoperative period (S2 Table). Additionally, the rates of postoperative changes in the angle of deviation were almost parallel between the two groups, which showed a continuous decrease after surgery (Fig 3A). This result corresponds with the study by Lee *et al*[24], who showed that the yearly amount of the exodrift decreased and was smallest between the fourth year and the last follow-up. When focusing on the overcorrected patients at 1 month after surgery, the postoperative angle of deviation also showed a continuous decrease after surgery (Fig 3B). However, in these patients, the exodrift rate between 1 and 6 months after surgery was significantly higher in the original RR group (*P* = 0.023). This was possibly due to the two cases with extremely large angles of esotropia in the original RR group that underwent reoperation for overcorrection at 6 and 7 months after surgery, respectively.

Kaplan-Meyer survival curves (Fig 2) also showed interesting results. The original RR group showed a continuous increase in recurrence over time, whereas the augmented RR group showed a first peak of recurrence around postoperative 1 year and a second peak around postoperative 3 years. Larger studies are needed to verify the difference of early and late recurrences.

Overcorrection rate was significantly higher in the augmented RR group from 1 to 6 months after surgery, which was predictable before the study. However, this difference kept decreasing throughout time, and finally overcorrection rates were comparable between the two groups at postoperative 2 and 3 years. There were 4 patients (6.3%) in the original RR group and 8 patients (14.0%) in the augmented RR group who were overcorrected at 2 years after surgery, which was not significantly different (*P* = 0.153). Two patients (3.1%) in the original RR group and no patient in the augmented RR group underwent reoperation for overcorrection. All the 12 patients showed overcorrection from 1 month after surgery, and most of their esodeviation showed a tendency to decrease over time or were stable, except for one patient whose esodeviation suddenly increased between 2 and 3 years after surgery (S3 Table). The overcorrection rate of unilateral RR procedure at postoperative 2 years has been reported to be 0% to 9.7%.[3, 5–7, 23] Our results, both in the original RR group and the augmented RR group, were comparable to those results, suggesting that the augmentation did not cause significantly more overcorrection than the original RR procedure.

This study has a few limitations. First, the data in this study was retrospectively collected. Since patients with good postoperative status are likely to be missed at follow-up examinations, long-term success rates could be underestimated. Survival analyses could have a selection bias resulting in underestimation or overestimation of long-term success rates. Second, the two surgical groups underwent surgery at different periods of time and patients were not randomly allocated in the original or augmented surgery groups. Original RR group was randomly selected from intermittent exotropia subjects over 3 years of age, who underwent surgery between February 2005 and August 2008. Augmented surgery was selectively performed after 2012 in intermittent exotropia subjects over 3 years of age. However, as nothing else changed, such as surgical instruments or suturing techniques during the whole period, it is reasonable to compare the results of these two procedures. Surgical skills of the surgeon could be different at those periods and this could affect the results. But taking into consideration that the surgeon had over 25 years of experience, difference of surgical skills can be ignored. Criteria for reoperation were identical between two groups. Third, our results cannot be applied to patients with large angles of exodeviation who were excluded from the study.

In conclusion, the long-term success rates were similar between the original RR group and augmented RR group in children with intermittent exotropia. The overcorrection rate was higher in the augmented RR group until 6 months after surgery, but no significant difference was found after 2 years. Since the long-term recurrence rate of the augmented RR group is significantly lower than the original RR group and this difference increased over time, higher success rates may be expected after a longer follow-up period. A larger prospective study with long-term follow-up is required to verify this concept.

## Supporting information

S1 Table Surgical outcomes: Original RR vs. augmented RR. (DOCX)

S2 Table Exodrift and recurrence rates. (DOCX)

S3 Table Patients with consecutive esotropia at 2 years after surgery. (DOCX)

## References

[1] Jeoung JW, Lee MJ, Hwang JM. Bilateral lateral rectus recession versus unilateral recess-resect procedure for exotropia with a dominant eye. American journal of ophthalmology. 2006;141(4):683–8. doi: 10.1016/j.ajo.2005.11.021 . 16564803 10.1016/j.ajo.2005.11.021

[2] Chia A, Seenyen L, Long QB. Surgical experiences with two-muscle surgery for the treatment of intermittent exotropia. Journal of AAPOS: the official publication of the American Association for Pediatric Ophthalmology and Strabismus / American Association for Pediatric Ophthalmology and Strabismus. 2006;10(3):206–11. doi: 10.1016/j.jaapos.2005.11.015 . 16814171 10.1016/j.jaapos.2005.11.015

[3] Maruo T, Kubota N, Sakaue T, Usui C. Intermittent exotropia surgery in children: long term outcome regarding changes in binocular alignment. A study of 666 cases. Binocul Vis Strabismus Q. 2001;16(4):265–70. . 11720592

[4] Yang M, Chen J, Shen T, Kang Y, Deng D, Lin X, et al Clinical Characteristics and Surgical Outcomes in Patients With Intermittent Exotropia: A Large Sample Study in South China. Medicine. 2016;95(5):e2590 doi: 10.1097/MD.0000000000002590 . 26844467 10.1097/MD.0000000000002590 PMC4748884

[5] Choi J, Chang JW, Kim SJ, Yu YS. The long-term survival analysis of bilateral lateral rectus recession versus unilateral recession-resection for intermittent exotropia. American journal of ophthalmology. 2012;153(2):343–51 e1. doi: 10.1016/j.ajo.2011.06.024 . 21982103 10.1016/j.ajo.2011.06.024

[6] Suh SY, Choi J, Kim SJ. Comparative study of lateral rectus recession versus recession-resection in unilateral surgery for intermittent exotropia. Journal of AAPOS: the official publication of the American Association for Pediatric Ophthalmology and Strabismus / American Association for Pediatric Ophthalmology and Strabismus. 2015;19(6):507–11. doi: 10.1016/j.jaapos.2015.08.011 . 26691028 10.1016/j.jaapos.2015.08.011

[7] Yang X, Man TT, Tian QX, Zhao GQ, Kong QL, Meng Y, et al Long-term postoperative outcomes of bilateral lateral rectus recession vs unilateral recession-resection for intermittent exotropia. International journal of ophthalmology. 2014;7(6):1043–7. doi: 10.3980/j.issn.2222-3959.2014.06.25 . 25540763 10.3980/j.issn.2222-3959.2014.06.25 PMC4270974

[8] Heo H, Sung MS, Park SW. Surgical outcomes of symmetric and asymmetric surgery for intermittent exotropia with postoperative large early overcorrection. Japanese journal of ophthalmology. 2013;57(5):475–80. doi: 10.1007/s10384-013-0260-x . 23832497 10.1007/s10384-013-0260-x

[9] Oh JY, Hwang JM. Survival analysis of 365 patients with exotropia after surgery. Eye. 2006;20(11):1268–72. doi: 10.1038/sj.eye.6702091 . 16167074 10.1038/sj.eye.6702091

[10] Choi J, Kim SJ, Yu YS. Initial postoperative deviation as a predictor of long-term outcome after surgery for intermittent exotropia. Journal of AAPOS: the official publication of the American Association for Pediatric Ophthalmology and Strabismus / American Association for Pediatric Ophthalmology and Strabismus. 2011;15(3):224–9. doi: 10.1016/j.jaapos.2010.12.019 . 21665502 10.1016/j.jaapos.2010.12.019

[11] Leow PL, Ko ST, Wu PK, Chan CW. Exotropic drift and ocular alignment after surgical correction for intermittent exotropia. Journal of pediatric ophthalmology and strabismus. 2010;47(1):12–6. doi: 10.3928/01913913-20100106-04 . 20128548 10.3928/01913913-20100106-04

[12] Kim H, Yang HK, Hwang JM. Long-Term Surgical Outcomes of Augmented Bilateral Lateral Rectus Recession in Children With Intermittent Exotropia. American journal of ophthalmology. 2016;163:11–7. doi: 10.1016/j.ajo.2015.12.003 . 26685790 10.1016/j.ajo.2015.12.003

[13] Lee SY, Hyun Kim J, Thacker NM. Augmented bilateral lateral rectus recessions in basic intermittent exotropia. Journal of AAPOS: the official publication of the American Association for Pediatric Ophthalmology and Strabismus / American Association for Pediatric Ophthalmology and Strabismus. 2007;11(3):266–8. doi: 10.1016/j.jaapos.2007.02.014 . 17572342 10.1016/j.jaapos.2007.02.014

[14] Yang HK, Hwang JM. Surgical outcomes in convergence insufficiency-type exotropia. Ophthalmology. 2011;118(8):1512–7. doi: 10.1016/j.ophtha.2011.01.004 . 21474185 10.1016/j.ophtha.2011.01.004

[15] Wang B, Wang L, Wang Q, Ren M. Comparison of different surgery procedures for convergence insufficiency-type intermittent exotropia in children. The British journal of ophthalmology. 2014;98(10):1409–13. doi: 10.1136/bjophthalmol-2013-304442 . 24842862 10.1136/bjophthalmol-2013-304442

[16] Kim C, Hwang JM. 'Largest angle to target' in surgery for intermittent exotropia. Eye. 2005;19(6):637–42. doi: 10.1038/sj.eye.6701604 . 15688062 10.1038/sj.eye.6701604

[17] Wright K. Color atlas of strabismus surgery. 2nd ed: Wright Publishing; 2000.

[18] Adams WE, Leske DA, Hatt SR, Holmes JM. Defining real change in measures of stereoacuity. Ophthalmology. 2009;116(2):281–5. doi: 10.1016/j.ophtha.2008.09.012 . 19091410 10.1016/j.ophtha.2008.09.012 PMC3903340

[19] Edelman PM BM, Murphree AL, Wright KW. Consecutive esodeviation, then what? Am Orthop J. 1988;38:111–6.

[20] Lee EK, Hwang JM. Prismatic correction of consecutive esotropia in children after a unilateral recession and resection procedure. Ophthalmology. 2013;120(3):504–11. doi: 10.1016/j.ophtha.2012.08.026 . 23174395 10.1016/j.ophtha.2012.08.026

[21] Richard JM, Parks MM. Intermittent exotropia. Surgical results in different age groups. Ophthalmology. 1983;90(10):1172–7. . 6657192

[22] Kushner BJ. Selective surgery for intermittent exotropia based on distance/near differences. Arch Ophthalmol. 1998;116(3):324–8. . 9514485 10.1001/archopht.116.3.324

[23] Lim SH, Hong JS, Kim MM. Prognostic factors for recurrence with unilateral recess-resect procedure in patients with intermittent exotropia. Eye. 2011;25(4):449–54. doi: 10.1038/eye.2011.12 . 21311571 10.1038/eye.2011.12 PMC3171229

[24] Lee JY, Ko SJ, Baek SU. Survival analysis following early surgical success in intermittent exotropia surgery. International journal of ophthalmology. 2014;7(3):528–33. doi: 10.3980/j.issn.2222-3959.2014.03.26 . 24967204 10.3980/j.issn.2222-3959.2014.03.26 PMC4067672

