# Supplementary material for: Long-term outcomes of augmented unilateral recess-resect procedure in children with intermittent exotropia
Source: PLoS One. 2017 Oct 6;12(10):e0184863. doi: 10.1371/journal.pone.0184863 (PMC5630122; doi:10.1371/journal.pone.0184863)
Supplement: S1 Table — (DOCX) [file pone.0184863.s001.docx]

| **Supporting Information**  **S1 Table.** **Surgical Outcomes: Original RR vs. Augmented RR** | | | | | | | | | | | | | |
| --- | --- | --- | --- | --- | --- | --- | --- | --- | --- | --- | --- | --- | --- |
|  |  | Success | | | | Recurrence | | | | Overcorrection | | | |
| Postoperative period | | 1 Mo | 6 Mo | 2 Yr | 3 Yr | 1 Mo | 6 Mo | 2 Yr | 3 Yr | 1 Mo | 6 Mo | 2 Yr | 3 Yr |
| Original RR (n=64, 59*) | No. | 53 | 51 | 47 | 36 | 1 | 7 | 13 | 20 | 10 | 6 | 4 | 3 |
|  | % | 82.8 | 79.7 | 73.4 | 61.0 | 1.6 | 10.9 | 20.3 | 34.0 | 15.6 | 9.4 | 6.3 | 5.1 |
| Augmented  RR (n=57, 49*) | No. | 26 | 38 | 45 | 36 | 1 | 1 | 4 | 7 | 30 | 18 | 8 | 6 |
|  | % | 45.6 | 66.7 | 79.0 | 73.5 | 1.8 | 1.8 | 7.0 | 14.3 | 52.6 | 31.6 | 14.0 | 12.2 |
| P-value |  | <0.001^a^ | 0.105^a^ | 0.479^a^ | 0.172^a^ | 1.000^b^ | 0.065^b^ | 0.036^a^ | 0.019^a^ | <0.001^a^ | 0.002^a^ | 0.153^a^ | 0.180^a^ |

^a^Chi-square test, ^b^Fisher's exact test, *number of patients examined at 3 years after surgery, RR = lateral rectus recession and medial rectus resection, No. = numbers, Mo = months, Yr = years
