# Supplementary material for: Long-term outcomes of augmented unilateral recess-resect procedure in children with intermittent exotropia
Source: PLoS One. 2017 Oct 6;12(10):e0184863. doi: 10.1371/journal.pone.0184863 (PMC5630122; doi:10.1371/journal.pone.0184863)
Supplement: S2 Table — (DOCX) [file pone.0184863.s002.docx]

| **S2 Table.** **Exodrift and Recurrence rates** | | | | |
| --- | --- | --- | --- | --- |
|  |  | Original RR | Augmented RR | P-value |
| Exodrift rate (PD/year) |  |  |  |  |
| Postop 1 ~ 24 months | Distance | 2.8 ± 3.4 | 3.0 ± 4.0 | 0.716^a^ |
|  | Near | 2.6 ± 3.6 | 2.8 ± 4.2 | 0.867^a^ |
| Postop 1 ~ 6 months | Distance | 8.1 ± 17.2 | 6.2 ± 19.5 | 0.588^a^ |
|  | Near | 6.5 ± 15.8 | 5.3 ± 25.5 | 0.760^a^ |
| Postop 6 ~ 24 months | Distance | 2.7 ± 3.4 | 3.0 ± 4.0 | 0.499^a^ |
|  | Near | 3.4 ± 7.1 | 2.6 ± 8.1 | 0.792^a^ |
| Postop 24 ~ 36 months | Distance | 1.9 ± 4.8 | 1.7 ± 6.0 | 0.863^a^ |
|  | Near | 1.5 ± 4.0 | 1.5 ± 5.9 | 0.967^a^ |

^a^Independent t-test, PD = prism diopters, RR = lateral rectus recession and medial rectus resection
