# Supplementary material for: Long-term outcomes of augmented unilateral recess-resect procedure in children with intermittent exotropia
Source: PLoS One. 2017 Oct 6;12(10):e0184863. doi: 10.1371/journal.pone.0184863 (PMC5630122; doi:10.1371/journal.pone.0184863)
Supplement: S3 Table — (DOCX) [file pone.0184863.s003.docx]

| **S3 Table.** **Patients with consecutive esotropia at 2 years after surgery** | | | | | | | |
| --- | --- | --- | --- | --- | --- | --- | --- |
| Age at surgery (years) / Sex | Preoperative angle of deviation (PD): Distance / Near | Surgery group | Angle of deviation at 1 Mo (PD): Distance / Near | Angle of deviation at 6 Mo (PD): Distance / Near | Angle of deviation at 2 Yr (PD): Distance / Near | Angle of deviation at 3 Yr (PD): Distance / Near | Reoperation for consecutive esotropia |
| 10 / F | 35 / 35 XT | Original | 12 / 12 ET | ortho / ortho | 12 / 10 ET | 6 / 6 ET | - |
| 3 / M | 35 / 25 XT | Original | 20 / 35 ET | 25 / 25 ET | - | - | RMR recession |
| 7 / F | 25 / 25 XT | Original | 40 / 35 ET | 39 / 31 ET | - | - | LMR recession |
| 7 / F | 40 / 40 XT | Original | 8 / 6 ET | 8 / 8 ET | 14 / 14 ET | 12 / 12 ET | - |
| 4 / M | 20 / 20 XT | Augmented | 16/ 16 ET | 16 / 16 ET | 24 / 24 ET | 16 / 16 ET | - |
| 9 / M | 25 / 18 XT | Augmented | 25 / 25 ET | 34 / 32 ET | 16 / 16 ET | 16 / 16 ET | - |
| 6 / M | 25 / 40 XT | Augmented | 10 ET / ortho | 32 / 52 ET | 16 / 16 ET | 10 / 10 ET | - |
| 6 / M | 25 / 30 XT | Augmented | 25 / 30 ET | 16 / 16 ET | 16 / 16 ET | 10 / 10 ET | - |
| 9 / M | 27.5 / 27.5 XT | Augmented | 25 / 25 ET | 16 / 16 ET | 12 / 12 ET | 4 / 4 ET | - |
| 5 / M | 27.5 / 20 XT | Augmented | 12 / 16 ET | 16 / 16 ET | 10 / 16 ET | - | - |
| 6 / F | 30 / 30 XT | Augmented | 18 / 18 ET | 16 / 16 ET | 12 / 12 ET | 36 / 36 ET | - |
| 4 / F | 40 / 40 XT | Augmented | 12 /10 ET | 13 / 11 ET | 16 / 16 ET | 14 / 14 ET | - |

M = male, F = female, Mo = months, Yr = years, PD = prism diopters, XT = exotropia, ET = esotropia, RMR = right medial rectus muscle, LMR = left medial rectus muscle
